# The Role of Helminth Infection and Environment in the Development of Allergy: A Prospective Study of Newly-Arrived Ethiopian Immigrants in Israel

**DOI:** 10.1371/journal.pntd.0004208

**Published:** 2016-01-11

**Authors:** Miguel Stein, Zalman Greenberg, Mona Boaz, Zeev T. Handzel, Mesfin K. Meshesha, Zvi Bentwich

**Affiliations:** 1 Allergy and Clinical Immunology Unit, The E. Wolfson Medical Center, Holon, Israel; 2 Public Health Laboratories, Ministry of Health, Jerusalem, Israel; 3 Biostatistics Unit, The E. Wolfson Medical Center, Holon, Israel; 4 Pediatric Division, Kaplan Medical Center, Rehovot, Israel; 5 Center for Tropical Diseases and AIDS, and Faculty of Health Sciences, Ben Gurion University, Beer Sheva, Israel; Universidad Nacional Autónoma de México, MEXICO

## Abstract

Helminth infection may be protective against allergy and account for the low prevalence of allergy in developing countries. We studied prospectively the prevalence of allergy in Ethiopian immigrants with heavy helminth infection on arrival in Israel, and again after a year of adjustment to an urban industrialized setting, to explore the roles of helminth infection, changed environment and background immunity on the manifestations of allergy. 126 newly arrived Ethiopian immigrants were studied at baseline and 115 after a year of follow up in Israel. Allergic symptoms, Skin prick tests (SPT), Tuberculin (PPD) skin tests, stool and blood samples were obtained for determining parasites, blood IgE and eosinophil levels, respectively. Anti-helminthic therapy was offered to the entire infected individuals, but only 50/108 (46.3%) took the medication. At baseline, there was a significant negative association between helminth infection and allergy, 4/18 (22.2%) of uninfected participants were allergic compared to 7/108 (6.5%) of helminth-infected participants (p = 0.028), as well as between helminth infection and SPT reactivity, 12/18 (66.6%) of uninfected participants compared to 43/108 (39.8%) of helminth-infected participants (p = 0.033). After one year, a significant general increase in allergy and SPT was observed. While only 11/126 (8.7%) were allergic at baseline, 30/115 (26.1%) became allergic at follow-up (p<0.0001), and while 55/126 (43.7%) were SPT+ at baseline, 79/115 (68.7%) became SPT+ at follow-up (p<0.001). A twofold increase in allergen sensitization was also observed after one year in Israel, particularly for dust mites, grasses and olive tree (p<0.001). These results show that: a) Helminth infection is significantly associated with low allergy and low SPT reactivity; b) One year after immigration to Israel, allergy and SPT reactivity increased significantly in all immigrants; c) Higher increases in positive SPT and allergy were observed after a year in the group that remained infected with helminths, even though they had a lowered helminth load; d) The reasons for the increased allergy one year after immigration needs further investigation but probably reflects the combined influence of the decreased helminth load and novel environmental factors.

## Author Summary

The role of intestinal parasitic infections in allergy is of great interest since it has been suggested that they suppress the development of allergy. Urbanization has also been claimed to cause allergy because of air pollution and higher exposure to allergens. The present study took advantage of the special opportunity presented by the recent Ethiopian immigration to Israel in order to study this question. A cohort of newly arrived Ethiopian immigrants to Israel, highly infected with parasites, was studied prospectively for the presence of allergy and response to allergens on arrival and after a year of follow up in Israel. The results showed clearly a significant inverse association between the presence of parasitic infections and allergy on arrival, while after a year of living in Israel, accompanied by a lowered parasitic infection load, a general increase of allergy was observed in all immigrant groups, and not only in those that had parasitic infections on arrival. These results lend support to the suppressive effect of intestinal parasites on allergy, but suggest that additional factors, most probably environmental, also play a role in the generation of allergy.

## Introduction

Asthma and allergy are major public health problems that are increasing in prevalence throughout the world. It has long been observed that the prevalence of allergic phenomena is by far higher in the developed countries than in the developing countries [1,2]. Studies have also shown a positive association between allergy and affluence within particular ethnic groups, suggesting a prominent environmental role rather than genetic factors [3]. The so-called “hygiene hypothesis” which posits that allergic phenomena arise from the sanitized living conditions of the developed world has been one of the major theories to account for this remarkable difference in prevalence of allergy [4]. Multiple mechanisms may account for the hygiene hypothesis, but there is considerable evidence to suggest that helminth infection plays a central role [4–6].

The clinical evidence for the role of helminth infection in allergy has been mixed. Several studies, including two large studies in Ecuador and Gabon, showed a negative association between SPT reactivity and helminth infection [6–9]. Similarly, the same Gabonese study and a large study performed in Venezuela, showed increased SPT reactivity in individuals who received antihelminthic treatment [10,11]. Conversely, other studies have shown an increase in allergic phenomena associated with helminth infection and a decrease after deworming. These include a study performed in China which showed a positive association of childhood asthma with ascaris infection, and a study performed in Venezuela, which showed an improvement in asthma symptoms after deworming [12,13]. Overall, the evidence suggests that a significant relationship does exist between helminth infection and allergy but that this interaction is complex and likely depends on additional factors such as environment, chronicity of infection, parasite burden, and parasite species.

During the last three decades, over 60,000 Ethiopian Jews have immigrated from Ethiopia to Israel, primarily in two large airlifts: called "Operation Moses" in the mid-eighties and "Operation Solomon" in 1991. These immigrants came mostly from rural areas in Northern Ethiopia and after arrival in Israel, they underwent medical evaluations and treatment. On arrival of both waves of immigration, they were found to have a very high prevalence of helminth infections with a very low prevalence of allergy, while after a year in Israel, many of them developed allergy. One study documented the asthma prevalence in this population as 2.5% on arrival and 17% after 8–17 years in Israel [14]. Studies conducted in Ethiopia generally indicated a low prevalence of allergic disease with the exception of certain areas [15], varying prevalence in rural-urban areas [16] and even lower prevalence of allergy, in helminth infected people [17].

In the early 1990s, we published several studies describing the immune profile found in helminth infected newly-arrived Ethiopian immigrants to Israel, and its possible role in the pathogenesis of HIV infection [18–23]. As we have described, this immune profile was characterized by a dominant Th2-profile, with chronic immune activation, and anergy [18–20]. The present study investigated prospectively the prevalence of allergy in relation to helminth infection and a changing environment in a population of Ethiopian immigrants to Israel. The study was done during the years 1997–2001 (collecting data, interviews, laboratory analysis and testing of the individuals). We published preliminary data in 2 abstracts at international meetings [21–22].

## Methods

### Study population

In total, 126 participants were included in the study (66 male, 60 female, mean age 31.2 ± 14.5). All individuals were recruited within the first three months of their arrival in Israel. Individuals with tuberculosis, syphilis, or HIV infections were excluded from the study. All 126 study participants had a similar socioeconomic and nutritional background.

### Ethics statement

Subsequent to the approval of the trial by the Kaplan Medical Center's IRB, participants were recruited from three immigrant absorption centers in Israel. Individuals were selected at random from these centers and were included in the study following their written informed consent (with the help of a trained veteran Ethiopian community worker that translated the questions to Amharic and explained the aim of the research).

### Clinical and laboratory testing

The new immigrants were interviewed by the physician with the help of an experienced Ethiopian social worker that translated the questions and explained the examination and the treatments. Individuals that needed further investigations were referred to secondary and tertiary health care services. Blood was taken in the absorption centers, and in the hospital as needed.

#### Allergy symptoms questionnaire

A questionnaire about respiratory symptoms relating to asthma and allergic rhinitis was administered after translation to Amharic and explaining the nature of the study. The questionnaire was partially based on the International Union Against Tuberculosis and Lung Disease (IUATLD) questionnaire and the European Community Respiratory Health Survey [24]. It included questions about recent symptoms or treatments associated with asthma and allergic rhinitis.

#### Skin prick test (SPT)

All participants had skin prick test for ten common allergens (*Dermatophagoides pteronyssinus*, *Dermatophagoides farinae*, feather mix, cockroach, cat pelt, dog epithelium, grass pollens mix, weed mix, olive, and cypress trees) using allergen extracts from Bayer Corp. (Spokane, WA, USA). Histamine (1 mg/ml) and glycerinated saline solutions were used as positive and negative controls, respectively. Allergens were applied to the volar surface of each participant’s forearm, in accordance with standard SPT procedure [25]. Responses were measured at 15 minutes with a wheal diameter of 3 mm or more constituting a positive response, after subtraction of the saline negative control. All SPT examinations were administered and interpreted by the principal investigator.

#### Tuberculin test (PPD)

Mantoux skin test for delayed type hypersensitivity with intracutaneous injection (0.1 ml) of tuberculin (5 TU) into the volar surface of the forearm was administered by a trained nurse and interpreted after 48–72 hours. Results were recorded in millimeters (mm) of largest induration diameter, with a 10 mm induration constituting a positive response.

#### Helminthic load determinations

Determination of helminth infection status was achieved by quantitative parasite egg load in stool specimens. Stools were obtained in one to three samples, to get at least 1 cubic centimeter (approximate 1 g) of stool, keeping the samples in a refrigerator at 4°C and analyzed by direct visualization within 7–30 days. Stool examinations and egg counts were performed according to a modification of the Richie formalin-ether sedimentation method [26]. One sample was tested in each subject; samples were added to a tube containing 2 mL of media for centrifugation. The sediment was analyzed to obtain the helminths load (eggs/cm^3^ of stool). The Richie method was the best method used at that time; today formalin-ether is replaced with ethyl acetate. The exact sensitivity of this method is not known but estimated to be above 90% [27].

#### Peripheral blood tests

Peripheral blood specimens were taken for analysis, including complete blood count, total eosinophil count and total serum IgE levels. IgE levels were determined by a solid phase, two-site fluoroimmunometric assay (Delfia Total IgE kit, Pharmacia).

### Study design

**Fig 1:**
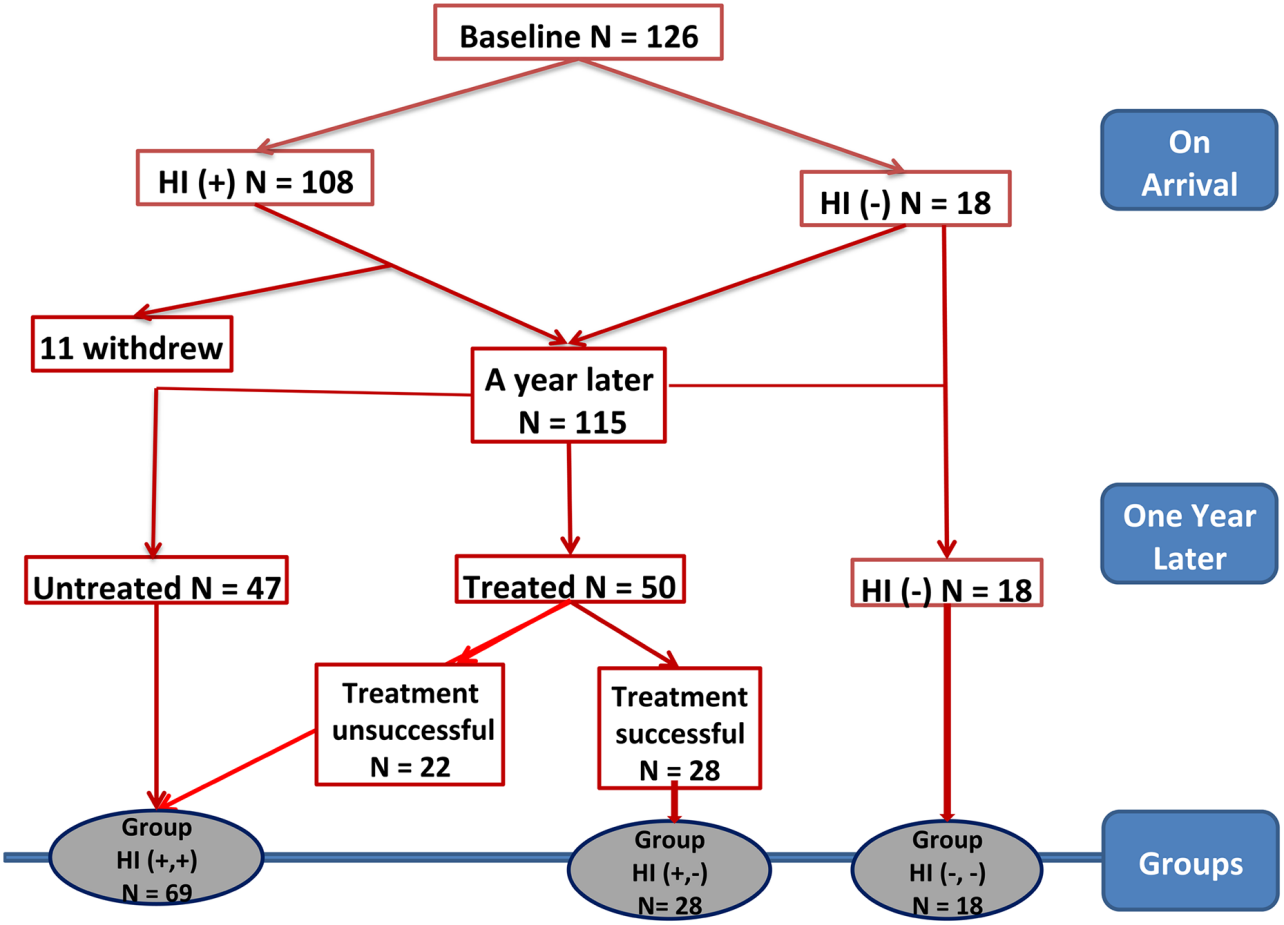
Flow chart of the study design. The groups are divided according to the status of helminth infection (HI) as helminth-infected (HI+) or helminth-uninfected (HI-) on arrival, and compared each individual between entry and after one year of follow up, then divided into three groups according to their HI status: persistent HI [HI(+,+) 69 individuals (60%)], eradicated HI [HI(+,-) 28 individuals (24.3%)], and those without HI [HI (-,-) 18 individuals (15.7%)].

All participants submitted to the above-mentioned clinical and laboratory tests at baseline and after one year. Allergic individuals were defined as those having both, positive SPT and clinical history of asthma and/or allergic rhinitis. We define allergy by the concomitant presence of positive SPT and clinical symptoms, since positive SPT alone, without clinical symptoms, is related to an atopic (sensitize) individual, not necessarily being allergic. Individuals with a negative clinical history of asthma or rhinitis, but with a positive SPT were defined as non-allergic SPT-positives (sensitized asymptomatic atopic individuals). Individuals with a negative clinical history and a negative SPT were defined as non-allergic normal individuals. The study design is shown in Fig 1. Regarding helminth infection, individuals were categorized as helminth-infected [HI(+)] or helminth-uninfected [HI(-)] based on the results of stool analysis.

Treatment of helminth infected individuals was offered for all the different kinds of helminths, the medications were given to the patients directly by the physician and/or by the health worker, together with a detailed explanation. As part of their ethnic/ cultural background, not all patients agreed to take the medications, and thus only 50 of the 108 infected individuals, received the therapy. *Schistosoma mansoni* was treated with praziquantel (40 mg/Kg divided in two doses for 1 day). Patients with *Hookworm*, *Ascaris*, and *Trichuris trichiura* infections, received albendazol (400 mg one dose) and or mebendazole (100 mg twice daily for 3 days, for Trichuriasis). Two individuals that were infected with *Strongyloides* received albendazole 400 mg twice daily for 3–7 days. *Taenia saginata* was found in 3 individuals and they received praziquantel.

### Data analysis

Statistical analysis was performed using SPSS v21 (IBM Inc, USA). This program provides p-values for chi square and Fisher’s exact test, depending on the expected number of values, and in all cases in which the expected value was < 5, the p-value for the Fisher’s exact test was utilized. Data were assessed for normality using the Kolmogorov-Smirnoff test. Continuous data were described using mean +/- standard deviation, while nominal data were described using frequency counts and are presented as n (%). Continuous data are compared by baseline helminth infection status using the t-test for independent samples or the Mann-Whitney U as appropriate. Changes from baseline to one-year data were compared using the t-test for paired observations or the Wilcoxon signed ranks test as appropriate. Nominal data were compared by helminth infection status at baseline using the chi square test, while baseline-to-one-year comparisons were made using the McNemar test. All tests are two-sided and considered significant at p<0.05.

## Results

### Helminth infection, allergy, and SPT at baseline

At baseline, helminthic infection was detected in 108 out of 126 (85.7%) individuals. *Hookworm* was the most prevalent species found in 79 individuals (73.1%), followed by *Schistosoma mansoni* in 51 (47.2%), *Ascaris* species in 50 (46.3%), and *Trichuris trichiura* in 16 individuals (14.8%). Multiple worm infection was found in 70 (64.8%) of the infected individuals. The mean stool helminths burden was 1264 ± 1950 eggs/cm^3^ (Table 1). Eosinophil counts were elevated across the entire cohort, regardless of helminths infection. However, HI(+) individuals had significantly higher levels of eosinophilia than HI(-) (781 ± 615 (median 657) versus 414 ± 334 (median 365) eosinophils/mm^3^, p = 0.01). Similarly, IgE levels were elevated across the entire cohort, and higher in the HI(+) group (1927 ± 1324 (median 1393) versus 1466 ± 1627 IU/ml (median 445), p = 0.06).

**Table 1:** Association between helminthic load and atopy in the new Ethiopian immigrants on arrival to Israel.

	*Allergic individuals	SPT (+)	** Non Allergic SPT(+)	***Normal individuals	Total
**# Individuals**	7 (6.5%)	43 (39.8%)	36 (33.3%)	65 (60.2%)	108 (100%)
**Helminth burden (eggs/cm**^**3**^ **of stool)**					
Average	384± 425	934± 1484	1041±1594	1482±2189	1264±1950
Median	240	262	333	424	333
Range	(10–1200)	(2–6000)	(2–6000)	(2–8252)	(2–8252)
Geometric Mean	190.5	211.7	216	291.4	256.6
**1 parasite (monoinfection)**	5 (4.6%)	20 (18.5%)	15 (13.9%)	18 (16.7%)	38 (35.2%)
**2 parasites**	1 (0.9%)	13 (12%)	12 (11.1%)	32 (29.6%)	45 (41.7%)
**3 parasites**	1 (0.9%)	9 (8.3%)	8 (7.4%)	14 (13%)	23 (21.3%)
**4 parasites**	0	1 (0.9%)	1 (0.9%)	1 (0.9%)	2 (1.9%)

SPT (+): Positive skin prick test to one or more allergen (includes the allergic and non-allergic, asymptomatic sensitized atopic individual).

* Clinically symptomatic individuals with asthma and/or rhinitis, with a positive skin prick test (SPT+).

** Asymptomatic individuals with SPT (+).

*** Asymptomatic and SPT negative individuals.

**Fig 2:**
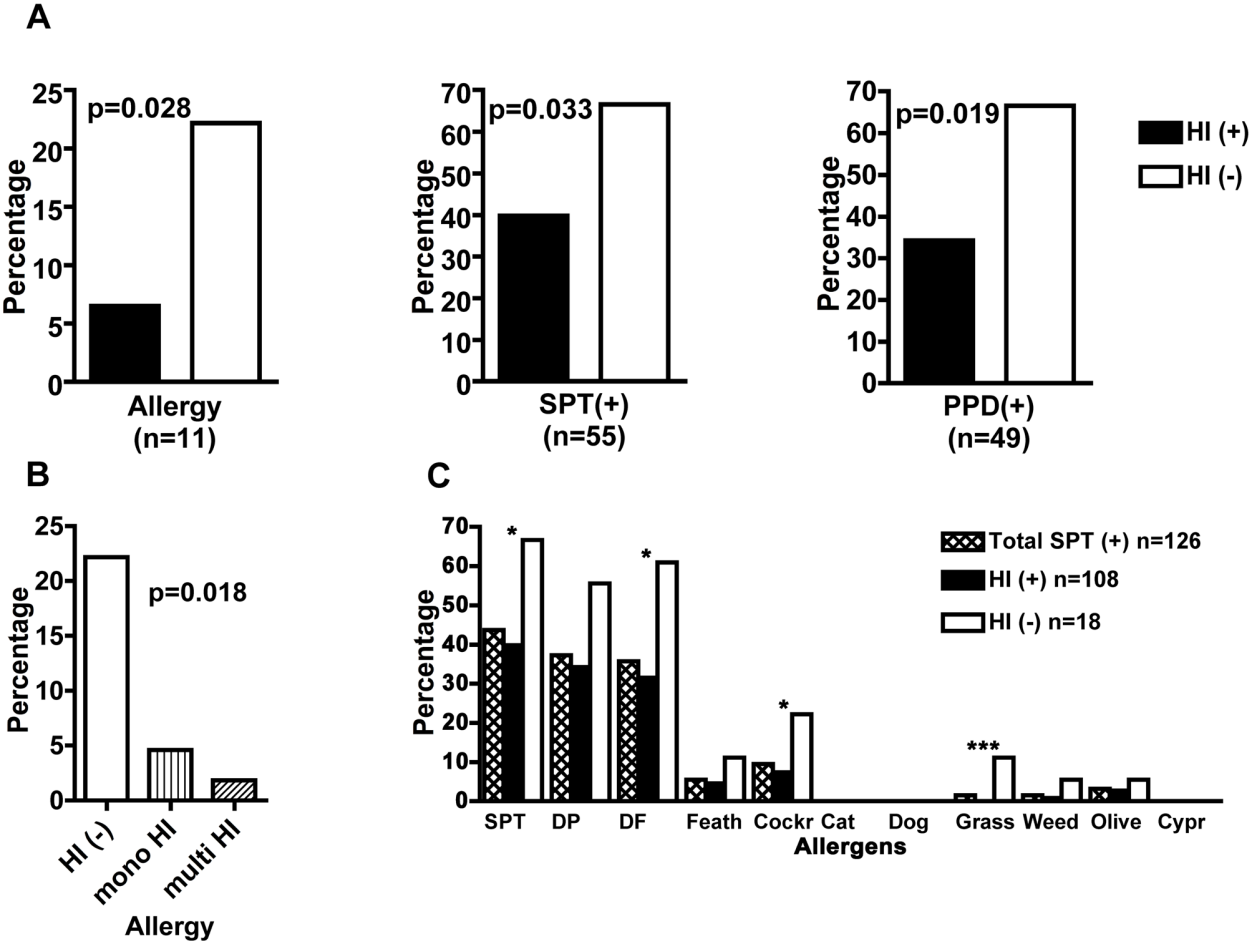
Helminth infections (HI) and the prevalence of allergy. Asthma and/or allergic rhinitis, positive skin prick test (SPT +) and delayed type hypersensitivity responses (PPD +) in the new Ethiopian immigrants on arrival to Israel. (A) The number of individuals in each group (n) and the statistical difference (p), between helminth infected (HI +) and non-infected (HI -) individuals. (B) The prevalence of allergic individuals at baseline and their HI status in absence of helminths (HI-) or in their presence (HI+), differences are shown for individuals with mono or multiple parasites infections. (C) The proportion of skin prick test (SPT) reactivity with the different allergens related to the HI status, is shown at baseline. Statistical p values <0.05 (*), <0.01 (**), and <0.001 (***). Chi square and Fisher’s exact test.

According to the allergic symptoms questionnaire and positive skin prick tests, allergy was present in 11/126 (8.7%) individuals at baseline. There were 3/126 (2.4%) individuals with clinical rhinitis but with negative SPT, who were not categorized as allergic individuals. Allergic individuals were predominantly male (9/11, p = 0.041) and older (mean age 41.2 ± 14.4 versus 29.7 ± 14.1, p = 0.01). The baseline association of helminth infection, allergy, SPT and PPD reactivity is depicted in Fig 2A and 2B. Allergen sensitizations and their association with HI, is shown in Fig 2C. There was a significant negative association between helminth infection and allergy such that only 7/108 (6.5%) of HI(+) participants were allergic compared with 4/18 (22.2%) of HI(-) participants (p = 0.028). Interestingly among HI(+) individuals, an inverse association was observed between allergy and the number of parasites, being 5/108 (4.6%) and 2/108 (1.9%) allergic individuals, if they were mono or multiple infected, respectively (p = 0.018, Fig 2B). Similarly, allergy was negatively associated with egg burden, such that the mean egg burden was lowest in allergic individuals, higher in non-allergic SPT-positives, and highest in non-allergic SPT negative normal individuals. The means of egg burden for these three groups were 384, 1041, and 1482 eggs/cm^3^, respectively (Table 1).

There was a significant negative association between helminth infection and SPT reactivity at baseline, such that 43/108 (39.8%) of HI(+) showed positive SPT reactivity compared with 12/18 (66.6%) of HI(-) individuals (p = 0.033, Fig 2A).

There was also a significant negative association between helminth infection and PPD skin test reactivity at baseline, such that 12/18 (66.6%) of HI(-) individuals were PPD-positive compared with 37/108 (34.3%) of the HI(+) participants (p = 0.019, Fig 2A).

### Helminth infection, allergy, and SPT at one-year

Out of the 126 original participants of the study, 115 individuals were available for follow-up at one year. The 11 individuals who withdrew from the study were all HI(+) and non-allergic, 5 of them being SPT-positive. Of the 115 participants available for follow-up, 97 (84.3%) were HI(+) at baseline. As part of their cultural ethnic background, not all the individuals agreed to take the medications, and so only 50 (46.3%) of the 108 infected individuals, received the antihelminthic treatment during the first 6 months of the study period. Of these fifty, 28 (56%) achieved successful eradication and 22 remained HI(+) at one year. Accordingly, the baseline HI(+) group was divided into two subgroups at follow-up: an HI(+,-) consisting of 28 individuals and an HI(+,+) group consisting of 69 individuals. The 18 individuals who began the study as HI(-), remained uninfected at follow-up. This group was defined as HI(-,-) for the purpose of before and after follow up analysis (the flow chart of the study groups is depicted in Fig 1). Peripheral blood eosinophilia did not change significantly, and total IgE increased slightly in the HI(+,+) group. It is worth noting that the mean egg burden in the HI(+,+) group decreased considerably from 1327 ± 1954 (median 435) to 699 ± 1345 (median 97) eggs/cm^3^ of stool, (p = 0.02).

**Fig 3:**
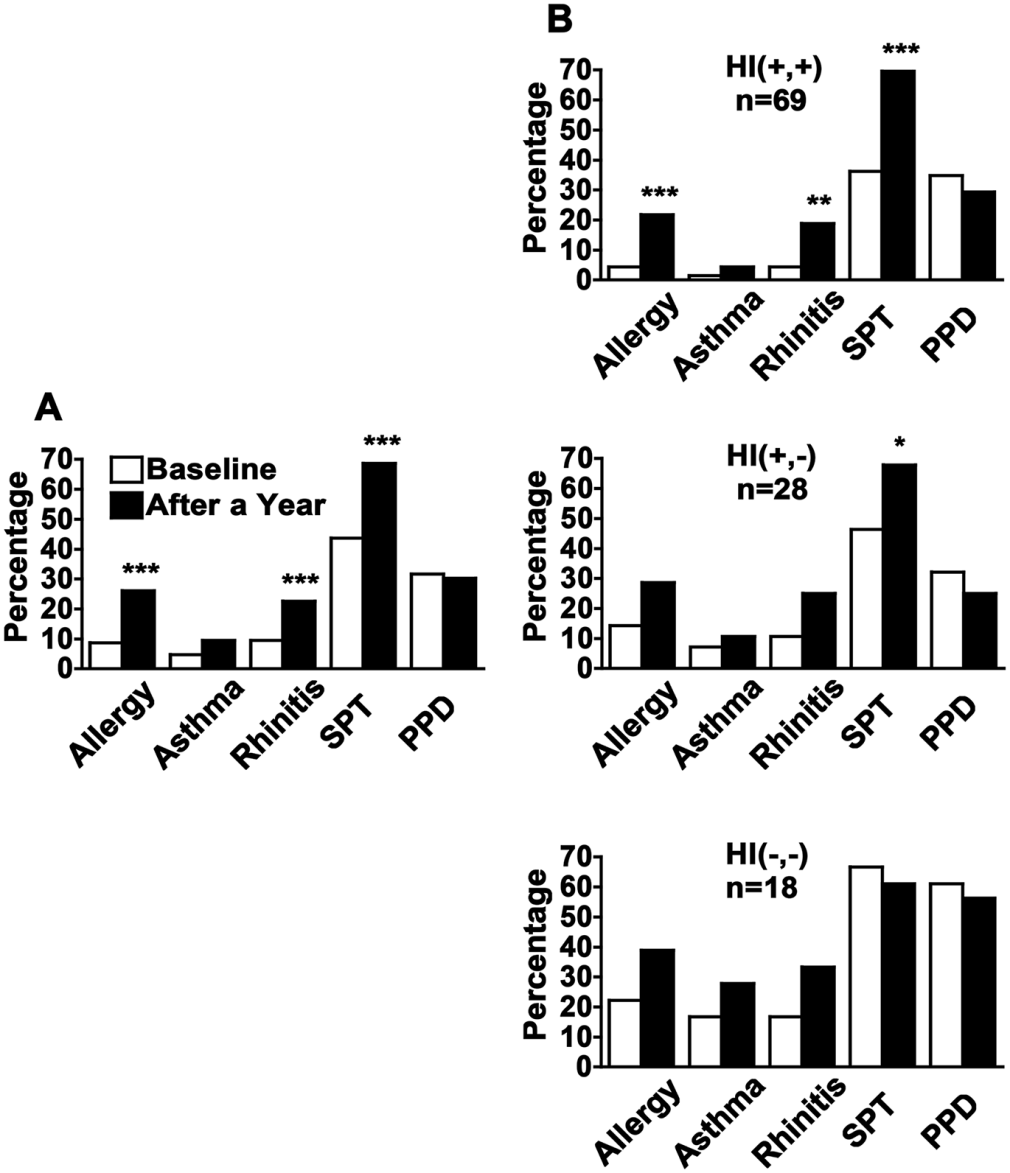
Allergy after one year. The prevalence of allergy (asthma and or allergic rhinitis), positive skin prick test (SPT) and delayed type hypersensitivity responses (PPD) in the whole cohort (A), and in the different groups (B), showing the association between helminth infections (HI) on arrival and after a year in Israel. Helminth infected individuals (n) were divided into three groups; HI (+,+): Individuals infected with helminths on arrival and in whom the infection persisted after a year, HI (+,-): Individuals infected with helminths on arrival that were not infected after a year, and HI (-,-): Individuals in whom no helminths were found on arrival and after a year. Statistical p values <0.05 (*), <0.01 (**), and <0.001 (***). McNemar’s test.

Allergy increased significantly in the overall cohort from 11/126 (8.7%) allergic individuals at baseline to 30/115 (26.1%) at follow-up (p<0.001, Fig 3A). Clinical history of allergy but with negative SPT was found in two individuals with rhinitis that were considered as non-allergic rhinitis patients. Asthma developed in 4.6% (5/115) of the individuals, and 13.9% (16/115) developed allergic rhinitis, being statistically significant only in the HI(+,+) group, such that 15.2% who were negative on the first exam were positive on the second exam (3/69 to 13/69, p = 0.002, Fig 3B). However, there was clearly a trend for increasing asthma and allergic rhinitis in the other groups as well (Fig 3B).

**Fig 4:**
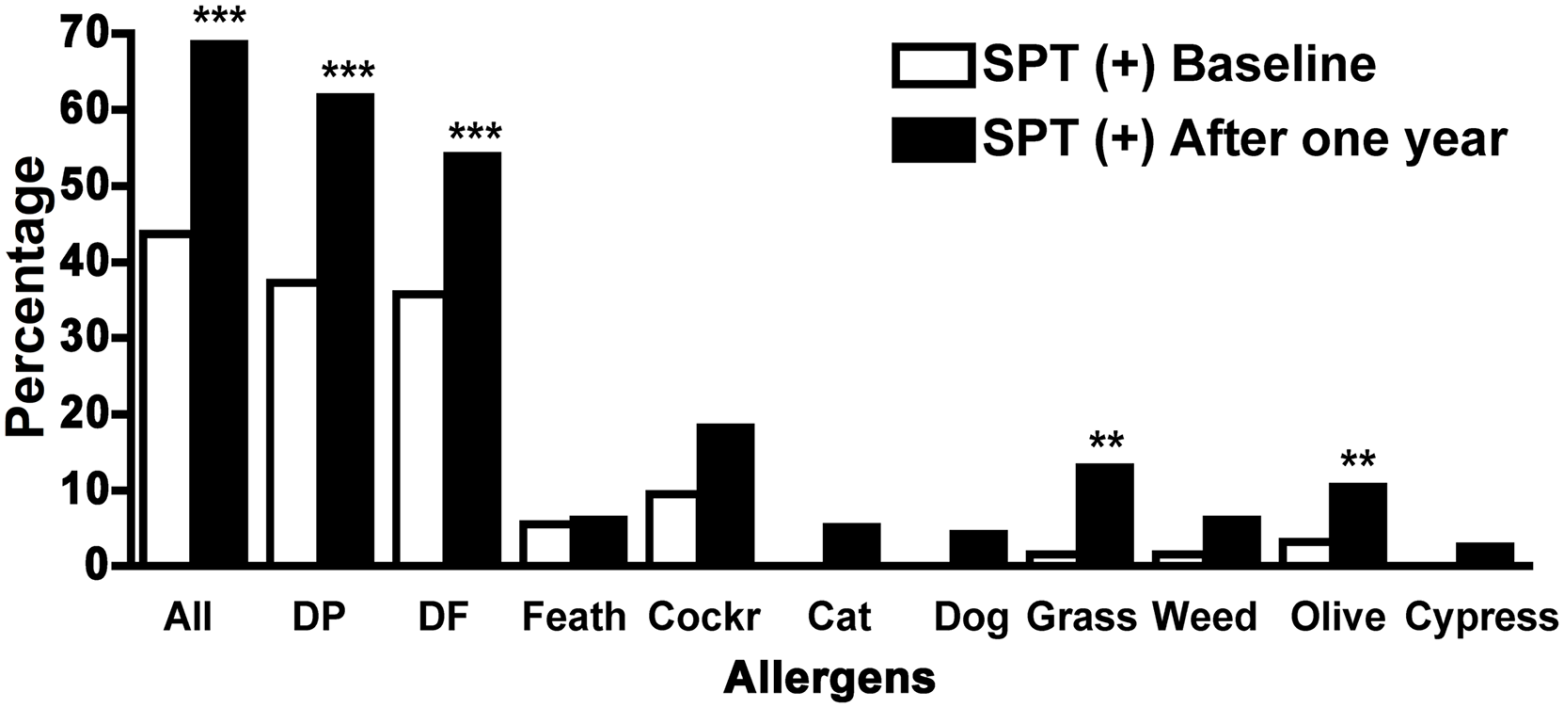
Response to allergens. Prevalence of allergen sensitizations by positive skin prick test (SPT +) to the different allergens are shown at baseline and after a year for the whole cohort. Dust mites *dermatophagoides pteronyssinus* (DP) and *dermatophagoides farina* (DF); feather mix (Feath); cockroach (Cockr); cat pelt; dog epithelium; pollens of grass mix; weed mix, olive and cypress trees. Statistical p values <0.05 (*), <0.01 (**), and <0.001 (***). McNemar’s test.

SPT reactivity also increased significantly in the overall cohort from 55/126 (43.7%) individuals at baseline to 79/115 (68.7%) at follow-up (p<0.001, Fig 3A), such that 27% (31/115) who were SPT negative at baseline became reactive after one year of follow up. Notably, significant changes in SPT reactivity were observed in the HI(+,+) and HI(+,-) subgroups, from 25/69 (36.2%) to 49/69 (71.0%) and from 13/28 (46.4%) to 19/28 (67.9%), respectively. Thus, 54.5% of the HI(+,+) and 40% of the HI (+,-) individuals who were SPT negative at baseline, became SPT positives on the second exam (p<0.001 and p = 0.03, respectively, Fig 3B). There were no significant changes in SPT reactivity in the HI(-,-) individuals [from 12/18 (66.7%) to 11/18 (61.1%)] nor in PPD reactivity–neither overall nor in subgroups. It is worth noting that in this paired analysis, the change of normal individuals at baseline that became sensitized but asymptomatic (SPT (+) non-allergic individuals) after a year, was significant for the groups that either remained infected or became non infected after eradication of the infection, as no changes were observed in the group without infection (p = 0.045 Table 2). Regarding specific allergens, a general increase in allergen reactivity with significant changes for dust mites, grasses and olive tree, was observed after a year of follow-up (Fig 4).

**Table 2:** Paired analysis of normal new Ethiopian immigrants on arrival, their association with helminths, and the development of new allergen sensitizations after one year in Israel.

Change from normal	HI (+,+) n (%)	HI (+,-) n (%)	HI (-,-) n (%)	P value
Normal to SPT (+)	24 (30.4%)	6 (20.7%)	1 (5.6%)	0.075
Normal to allergic*	5 (6.3%)	2 (6.9%)	1 (5.6%)	0.98
Normal to non-allergic SPT (+)**	**19 (24.1%)**	**4 (13.8%)**	**0**	**0.045**

SPT (+): Positive skin prick test to one or more allergen (includes the allergic and non-allergic, asymptomatic sensitized atopic individual).

* Clinically symptomatic individuals with asthma and/or rhinitis, with a positive skin prick test (SPT+).

** Asymptomatic sensitized atopic individuals with SPT (+).

HI (+,+): 79 individuals infected with helminths on arrival and in whom the infection persisted after a year. 2^nd^ SPT was not done in 10 individuals.

HI (+,-): 29 individuals infected with helminths on arrival that were not infected after a year. 2^nd^ SPT was not done in 1individual.

HI (-,-): 18 individuals in whom no helminths were found on arrival and after a year.

P value: Chi square and Fisher’s exact test.

**Fig 5:**
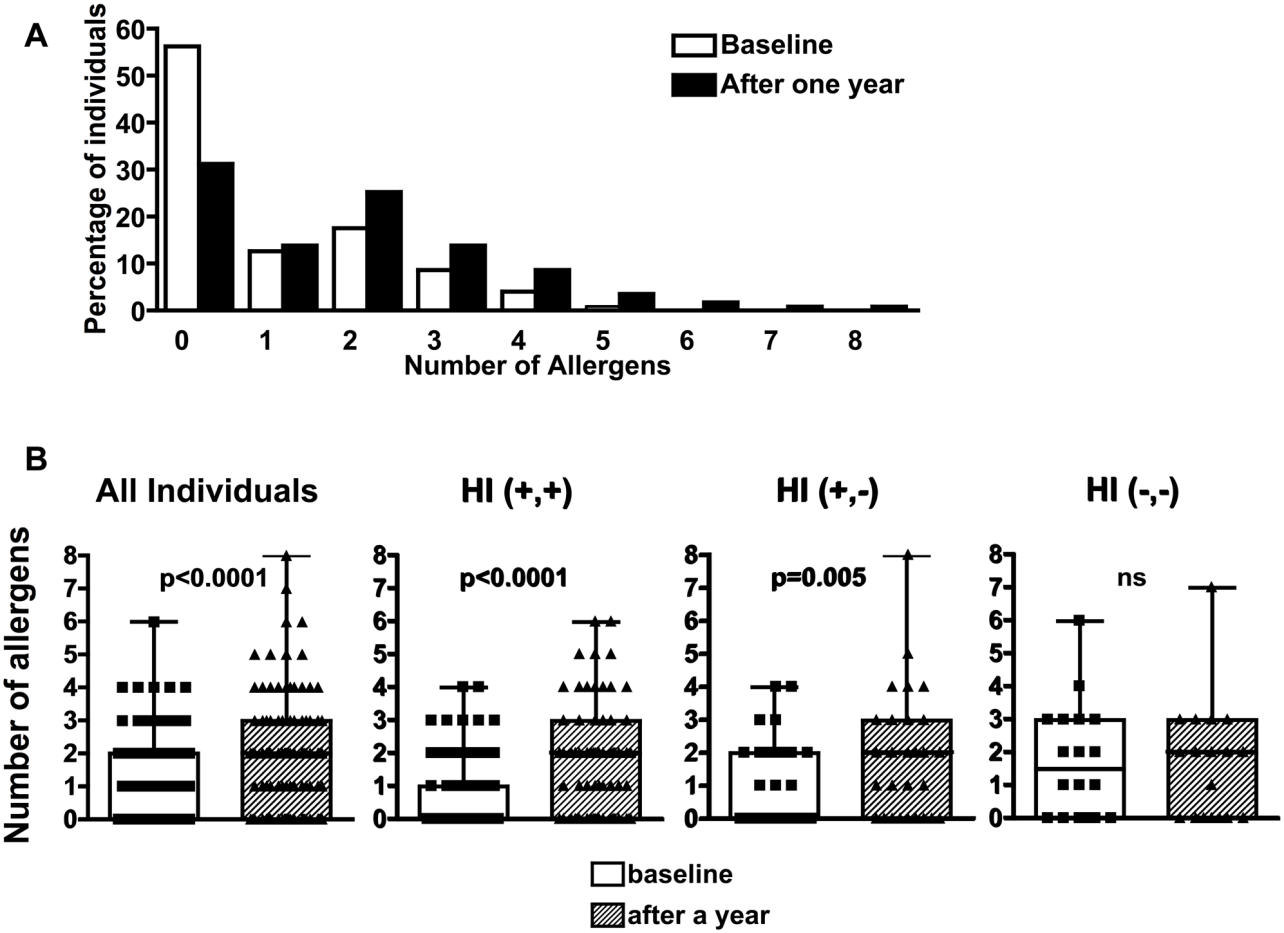
Individual allergen sensitization. (A) Proportion of individuals reacting between 0 and 10 different allergens (McNemar’s test) and (B) Number of allergen sensitizations per individual in the different helminth infections (HI) groups, at baseline and after a year. The distribution of the number of different allergens per individual causing positive SPT responses are represented by the boxes that represent 75% of the data values. The horizontal black line across the box marks the median value. The error bar shows the 90^th^ percentile of the population. Individual data-points falling beyond that boundary are shown as dots. HI (+,+): individuals with persistent infection after a year, HI (+,-): individuals infected with helminths on arrival and not infected after a year, and HI (-,-): individuals in whom no helminths were found on arrival and after a year. Statistical differences between the groups are shown, p values <0.05 (*), <0.01 (**), and <0.001 (***). ns: not significant. Wilcoxon signed ranks test.

Interestingly, the mean allergen sensitization increased by 2 fold, with an increase in the number of allergens sensitizations per individual after a year in all the population (Fig 5A and 5B). In a paired analysis, 31/115 (27%) individuals became sensitized to one or more allergens and 27/115 (23.5%) individuals increase their own previous numbers of allergens sensitization. At baseline, the median number of allergens detected by SPT per individual was 0 (range 0–6), that median number increased to 2 (range 0–8) allergens per individual after a year of follow-up (p<0.001). Furthermore, there were significant increases in the number of allergens sensitizations per individual, in the HI(+,+) and HI(+,-) groups but not in the HI (-,-) ones (p<0.001 and p = 0.005, respectively, Fig 5B).

## Discussion

The results of this study clearly demonstrate an inverse association between helminth infection and allergy in the newly arrived Ethiopian immigrants to Israel. After one year in Israel, a striking increase of allergy in all the cohort of immigrants was observed which may reflect a complex interaction of the host immune system with the changed burden of helminth infection and with the new environment.

Anti-helminthic therapy was offered to the entire infected individuals, but as part of their cultural ethnic background, not all the individuals agreed to take the medications. Thus only 50 of the 108 infected individuals (46.3%) took the medication. Not all the treated individuals eradicated their helminth infections, suggesting that some treated individuals will need a second course of therapy to eradicate their helminths. Overall, helminth infection burden decreased in infected individuals, after a year of follow-up.

High levels of eosinophils and Total IgE were observed on arrival on both infected and uninfected individuals, probably due to previous exposure and infections with helminths with the resultant chronic immune stimulation with a Th2 profile. The fact that the non-infected individuals had lower levels of eosinophils and IgE, certainly lends support to this interpretation. We have previously described that these changes will take several years to normalize, probably suggesting that the environment is the main factor affecting IgE levels and eosinophils in this population, though the possible contribution of genetic background cannot be ruled out [32,33].

Asthma and allergy are major public health problems that are increasing in prevalence in most developed countries. Allergic diseases are also expected to increase, as lifestyles become more urbanized or “westernized” in developing countries. However, the reasons for this increase are still poorly understood, especially in view of the striking negative association between allergy and a "dirty" environment. Furthermore, it has also been suggested that helminth infection may be protective against allergy and possibly autoimmune diseases in developing countries [28].

Reports on the role of helminth infection in allergy have been contradictory. Some studies showed that helminthic infection suppresses allergy and therefore treatment of helminthic infection may predispose to allergy (Reviewed by [29,30]), while other studies demonstrated that helminthic infection has no protective role and even enhances allergy[4,13,31]. In our study, helminth infection was clearly and significantly associated with low allergy and low SPT reactivity in the newly-arrived Ethiopian immigrants at baseline, which suggest that helminths are indeed protective against allergy. The lower helminths load analyzed by the burden of eggs/cm^3^ of stool or by the number of parasites affecting the individuals, was associated with a higher prevalence of SPT positivity. Infection with several parasites had even lower allergy than monoinfection compared to the non infected individuals. It is worth noting that heminth infected individuals had a significant decreased PPD reactivity on arrival. We couldn’t observe significant changes in PPD reactivity between the groups after a year of follow-up period. The persistent chronic immune activation and anergy can probably account for that [18–20]. SPT positivity demonstrates sensitization to allergens, and not necessarily clinically allergic individuals, indicating that there is an important group of asymptomatic sensitized individuals (Table 1). Furthermore, a significant number of normal individuals became sensitized without being clinically allergic individuals (Table 2).

Interestingly, we found that allergy and SPT increased significantly in all immigrants after one year in the new environment. The interpretation of this increase is not entirely clear. It may reflect a decrease in the worm infection load, which was observed in the dewormed group, but it could also reflect exposure to novel allergens, consistent with the significantly increased SPT reactivity to new aeroallergens such as grasses and olive tree. A recent study done in South America, demonstrated that children that improved their environmental conditions and had lowered their infectious burden, were more likely to have a responsive phenotype to produce Th1, Th2 and T regulatory cytokines after mitogen stimulation of whole blood, and that was associated with an increased prevalence of atopy [34].

The number of allergens sensitizations per individual increased in all the population after a year, being significant for the individuals that had either persistent or eradicated their helminths. Parasite infection can induce IgE antibodies that cross react with aeroallergens, this has been described for *Wuchereria bancrofti* for helminth glutathione-S transferase and the aeroallergen Bla g 5 from cockroach [35], and for tropomyosin from filarial and *ascaris* that can cross react with dust mites and cockroach [36,37]. Our study has not tried to address this possibility which could very well account for the increased allergic responses to the allergens prevalent in Israel.

Another possible explanation for the increased allergy observed in the immigrants after arriving in the new environment is a persistent skewed Th2 immune profile [20], induced by previous exposure to helminths, such persistent immune profile could make the immigrants more susceptible to allergy, after reducing their helminth load.

Taken together, the results of the present study suggest that helminth infection confers protection from allergy but also point to the possible role of the new environment and the change in the helminth load, in increasing allergy. Similar immigrations are occurring worldwide and therefore additional studies of such situations would be important in gaining a better understanding of these phenomena and will be of great importance to public health in the coming years. The growing efforts and investments to control and eradicate helminth infections in developing countries clearly present such situations that will be extremely important to study because of their possible impact on the occurrence and prevalence of allergy in these countries.

## Supporting Information

S1 Checklist Checklist for the management of study. (DOC)
